# Effect of processing parameters on texture and variant selection of as-built 300 maraging steel processed by laser powder bed fusion

**DOI:** 10.1038/s41598-022-19835-9

**Published:** 2022-09-28

**Authors:** Adriana Eres-Castellanos, Ana Santana, David De-Castro, Jose Antonio Jimenez, Rosalia Rementeria, Carlos Capdevila, Francisca G. Caballero

**Affiliations:** 1 MATERALIA Research Group, Department of Physical Metallurgy, National Center for Metallurgical Research (CENIM-CSIC), Avda. Gregorio del Amo 8, 28040 Madrid, Spain; 2 Department of Metallurgical and Materials Engineering, Colorado School of Mines, 920 15th St, Golden, 80401 USA; 3 ArcelorMittal Global R&D SLab—Steel Labs, Calle Marineros 4, 33490 Avilés, Spain

**Keywords:** Metals and alloys, Mechanical engineering

## Abstract

Among the materials that might be manufactured with laser powder bed fusion (LPBF), one can highlight maraging steels, with excellent weldability, strength and fracture toughness. However, the effects of the processing parameters and the mechanisms governing the as-built texture are not clear yet. A recent publication showed a low texture index in the prior austenite, in contrast to other alloys subjected to LPBF with the same strategy. Authors suggested several hypotheses, although no conclusions were drawn. This work aims to investigate these findings by using a 300 maraging steel processed under different conditions, i.e. different printer, powder layer thickness and laser emission mode. To do so, X-Ray Diffraction, Electron Backscattered Diffraction and Scanning Electron Microscopy have been used. Results show that the heat treatment intrinsic to the LPBF process does not affect the prior austenite grains, whose texture and morphology remain unchanged throughout the process. Also, for the studied ranges, the microstructure texture is not related to the powder layer thickness or to the laser emission mode, although it could be affected by the laser power or the scan strategy. Finally, a low degree of variant selection has been observed, where the selected variants are those that contribute to a martensite cubic rotated texture.

## Introduction

Additive manufacturing (AM), commonly known as 3D printing, is a manufacturing process consisting of incremental layer-by-layer deposition, melting, fusion and binding of the material^1^. Among its benefits, one can highlight the possibility to manufacture complex parts at once, using an optimum amount of material^2^. Among the different types of AM processes for metals, some of the most important ones are based on powder bed fusion: laser powder-bed fusion (LPBF) and electron beam melting (EBM)^3^.

In LPBF, a powder layer of a given thickness is deposited on top of previously melted layers. Subsequently, the layer is melted and fused to the previously melted layers by using a laser^3^ characterized by several parameters, such as power, speed, beam diameter, wavelength or emission mode. An optimum selection of the process parameters can help to reduce the porosity of the final structure, thus improving the part mechanical properties^4^. Many scanning options are provided in commercial LPBF machines, where the most used one is probably the hatch strategy^5^. During hatching, the laser typically moves with a given speed along parallel lines, whose direction is called scan direction (SD). The distance between them is named hatch spacing and the direction perpendicular to the deposition sections is called building direction (BD). The rotation of the SDs on successive layers is a common strategy, where the rotation of 67° (hatch angle) has been proposed to maximize the number of layers with different SDs^6^. Commercial LPBF machines also offer different types of laser emission mode, as previously mentioned. According to the laser emission mode, lasers can be continuous wave (CW) emission or pulsed wave (PW) emission. CW emission lasers emit continuous, constant intensity radiation, while PW emission lasers emit regularly spaced and very short light pulses. Because of their continuous character, CW emission lasers create elongated melt pools (MPs), which are called tracks. On the other hand, PW emission lasers lead to groups of MPs which can be superposed to each other. The PW emission mode parameters are: point distance (distance between adjacent MPs), exposure time (time that the laser is stopped at a given point, while turned on) and jump delay (time during which the laser is turned off while moving to the next point). For short exposure times and long jump delays, PW emission lasers are attributed to lead to faster solidification rates and to avoid heating, which minimizes the thermal distortion^7^.

Although non-ferrous alloys have been firstly envisioned as perfect candidates for LPBF process, the study of the most successful family of alloys, steels, processed by this technique, is not far behind^8^. Among steels, one can highlight maraging steels, characterized by very low carbon contents and by very high fractions of substitutional elements to be precipitated during a subsequent aging treatment^9^. Their outstanding weldability and mechanical properties (ultra-high strength and fracture toughness) make them ideal for applications that require high strength-to-weight ratios, such as landing gears and slat tracks for the aerospace industry, as well as high-performance parts in the power plant and injection molding industries^8^.

Grade 300 maraging steels are the most widely used maraging steels in AM and have shown a different resultant microstructure^8^ than the one obtained by conventional processing, with comparable mechanical properties^10^. However, one cannot neglect the crystallographic anisotropic nature of the LPBF process, associated to the solidification mode, i.e. cellular for maraging 300 steels^11,12^, and to the scanning strategy^13^. Crystallographic texture is directly related to some mechanical properties, reason why it is important to focus on this point. So far, studies have shown that a 90° scan strategy diminishes the degree of anisotropy in the martensitic structure, with respect to the non-rotation strategy^13^, because of the rotation of the heat flux direction^14^. In most of the studies on the texture of 300 maraging steels subjected to LPBF^13–15^, the texture of the parent austenite has not been discussed. In addition, in some cases, the martensitic texture has been assessed based on EBSD scans of small area, that might not have been representative of samples^13–15^. A recent publication by Kannan and Nandwana^16^ assessed the texture of the parent austenite and martensite, as well as the variant selection phenomenon, in the as-built microstructure of a 300 maraging steel subjected to LPBF with an unknown scan strategy, a laser power of ~ 110 W, a scan speed of ~ 1500 mm/s, a hatch spacing of ~ 50 µm and a layer thickness of ~ 45 µm. They showed that martensite did not present any predominant texture component or fiber in the as-built condition. They also concluded that the prior austenite showed a cube texture with minor fractions of rotated-Goss, although the texture index was low (maximum ODF intensity < 2 MRD), in contrast to other alloys subjected to LPBF, such as austenitic steels^17^. Kannan and Nandwana^16^ suggested several hypotheses that could explain the lack of prior austenite texture, i.e. (a) the intrinsic heat treatment during the printing process could have led to the recrystallization of the prior austenite structure; (b) the interaction of pores with the material during solidification could have led to the presence of randomly oriented austenite grains and (c) the random texture could be explained based on the thermal gradient and solidification velocity space, which are dependent on the processing parameters. Variant selection phenomena were reported to be negligible. This work aims to further investigate these findings and answer two questions: (a) can the resultant texture and variant selection be intimately associated to the processing parameters and how? And (b) how does the thermal flux and the heat treatment intrinsic to the LPBF process affect the prior austenite texture? To do so, a 300 maraging steel was processed by LPBF with a 67° scan strategy, where the printer, hatch spacing, layer thickness and the laser emission were varied to evaluate the effect of these parameters on the final microstructure. The microstructures were studied at different heights in terms of macro-texture and variant selection by X-Ray Diffraction and Electron Backscattered Diffraction. Detailed high magnification analyses with Scanning Electron Microscopy were included to further understand these solidification and transformation mechanisms.

## Methods

In this work, commercial Maraging 300 powder was used to build parts by LPBF. Parts built in Maraging 300 have a chemical composition corresponding to the United States classification 18% Ni Maraging 300. The steel chemical composition, relative density and density are included in Supplementary Material A.

Parts built by LPBF in an EOS M270 machine (cylinders with a height of 10 mm and a diameter of 6 mm) were used as a reference in this work. Printing was carried out under a N_2_ atmosphere, where the laser emission mode was CW, the volume rate (which is a function of the laser power and speed) was 3 mm^3^/s and the layer thickness was 40 μm. The hatch strategy of an individual layer consisted of a meander pattern with a 100 μm hatch spacing. Successive layers were rotated by a 67° angle.

Additional conditions studied in this work were built by a RENISHAW printer under an Ar atmosphere, with a laser power of 250 W and an average laser speed of 1000 mm/s. The hatch strategy of an individual layer consisted of a meander pattern with an 80 μm hatch spacing, where the SD was rotated by 67° between consecutive layers. Two parameters were systematically varied. The first of these parameters was the layer thickness, which took values of 50 and 100 μm. The second one was the laser emission mode, which was set as either CW or PW. The point distance, exposure time and delay jump values were 20 μm, 20 μs and 0 μs for the CW laser and 70 μm, 60 μs and 10 μs for the PW laser, respectively. In this case, built samples were square prisms with a height of 10 mm and a square side length of 10 mm and they were machined to obtain four samples with sections of 4 × 4 mm^2^. None of the conditions in this work presented significant porosity. From now onwards, the conditions are identified according to their printer, layer thickness and laser emission mode. A simplified representation of the evolution of the power and distance increase along a track as a function of time for the different printing conditions, as well as a sketch of the scan rotation, can be found in Supplementary Material B.

The first (bottom) and last (top) layers were subjected to X-Ray Diffraction texture measurements. To do so, both the bottom and the top layers were subjected to standard metallography procedures, followed by several etching and polishing cycles. XRD measurements were performed by a Bruker AXS D8 X-ray diffractometer, with a Co X ray tube working at 40 kV and 30 mA in parallel-beam geometry and equipped with a LynxEye Linear Position Sensitive Detector. Conventional diffraction patters were collected in Bragg–Brentano geometry over a 2θ range of 45°–135° with a step size of 0.01°. These XRD profiles were analyzed using the 4.2 version of the program TOPAS (Bruker AXS), identifying peaks of martensite ($\alpha ^{\prime}$) and retained austenite ($\gamma^{ + }$). Subsequently, three incomplete pole figures (PF), corresponding to the planes $( {2\; 0 \;0} )_{\alpha ^{\prime}}$, $( {2\; 1 \;1} )_{\alpha ^{\prime}}$ and $( {1\;1 \;0} )_{\alpha ^{\prime}}$, were measured in the back-reflection mode, using a pole distance in the range 0°–70°. In all cases, the use of a collimator of 1 mm diameter and a lineal detector centered at the 2θ position of these reflections enabled the collection of the whole diffracted intensity distributed over the angular range in the vicinity of the ideal focusing point. As the whole peak profile was covered at the ideal Bragg angle positions, the loss of intensity due to defocusing was compensated. On the other hand, the background contribution was eliminated using measurements far enough from the peak edge on the side of each reflection. From the experimental PFs, the orientation distribution function (ODF); was derived by the use of the de la Vallée Poussin method^18^ implemented in the MATLAB^®^ toolbox MTEX^19^, assuming a cubic lattice structure and a triclinic sample symmetry, and subsequently ghost corrected. With respect to the texture of $\gamma^{ + }$, the low intensities that were detected (volume fractions of maximum 7 ± 3%), did not enable to perform texture measurements. However, the $\gamma^{ + }$ texture was indirectly studied by comparing the measured diffractogram to the predicted one by the Rietveld approach without texture correction^20^.

The microstructures located in the middle, in both the transverse (T) and longitudinal (L) section of the sample built in the EOS M270 machine—approximate at a height of 5 mm—were also characterized by Electron Backscattered Diffraction (EBSD) in a Zeiss Auriga Compact Focused Ion Beam-Scanning Electron Microscope (FIB-SEM), operating at 20 kV. Supplementary Material B includes a sketch showing the location of these two sections. Two areas of 570 × 765 µm^2^ were scanned per section, with a step size of 1 µm. In all cases, only the martensitic structure was considered, since the retained austenite volume fraction was very low and it was difficult to index for that given step size. Both sections were subsequently analyzed at high magnification (74 × 80 μm^2^) by using a step size of 0.1 μm. Both bcc and fcc phases were considered at this magnification. EBSD data analyses were carried out by MATLAB^®^, specifically by its toolbox MTEX^19^.

SEM imaging, correlative to the high magnification EBSD scans, was performed after slightly polishing and etching the sample with a 2% Nital solution, by using a JEOL JSM-6500 FEG-SEM with a secondary electron detector. Because of the slight polishing and etching procedure and because of the fact that the EBSD scans are performed on a tilted surface, these SEM micrographs could be slightly distorted with respect to their corresponding maps.

## Results

**Figure 1:**
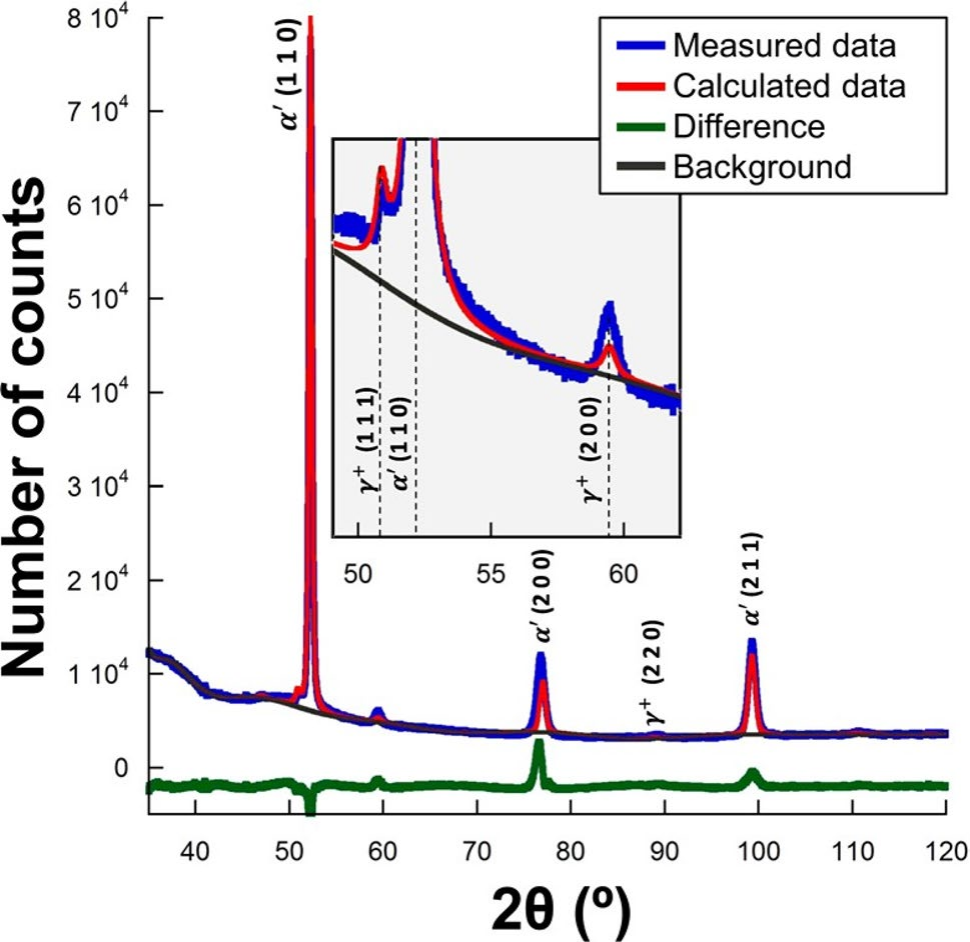
Example of measured XRD diffractogram (blue) obtained for the EOS—40 μm—CW laser condition, where peaks of martensite $\alpha ^{\prime}$ and retained austenite $\gamma^{ + }$ are identified. The diffractogram calculated by the Rietveld method without texture correction is shown in red, where the difference of this calculated diffractogram with respect to the measured one is shown in green and the background is shown in black.

As a first approach to evaluate the effect of the processing parameters on the texture of both $\alpha ^{\prime}$ and $\gamma^{ + }$, the XRD diffractograms were studied. Figure 1 shows in blue an exemplified XRD diffractogram collected from the EOS—40 μm—CW laser condition, where the martensite and retained austenite peaks were identified, although the volume percentage of the latter phase was rather low, i.e. < 7 ± 3%. The diffractogram calculated by the Rietveld method without texture correction is superimposed on the figure in red and the difference between both the measured and the calculated data is shown on the bottom in green. The discrepancies between the measured and calculated data are evident, denoting that both phases present texture. Especially, peaks corresponding to the (2 0 0) planes of both $\alpha ^{\prime}$ and $\gamma^{ + }$ that show significantly different values with respect to their measured intensities. This behavior was observed for all the studied conditions. This suggests that both phases present texture components or fibers where the $\langle {1\;0\;0} \rangle$ directions are parallel to the BD. However, as previously stated, the low volume fraction of $\gamma^{ + }$ did not enable to measure its texture, reason why XRD texture measurements were only performed in $\alpha ^{\prime}$.

**Figure 2:**
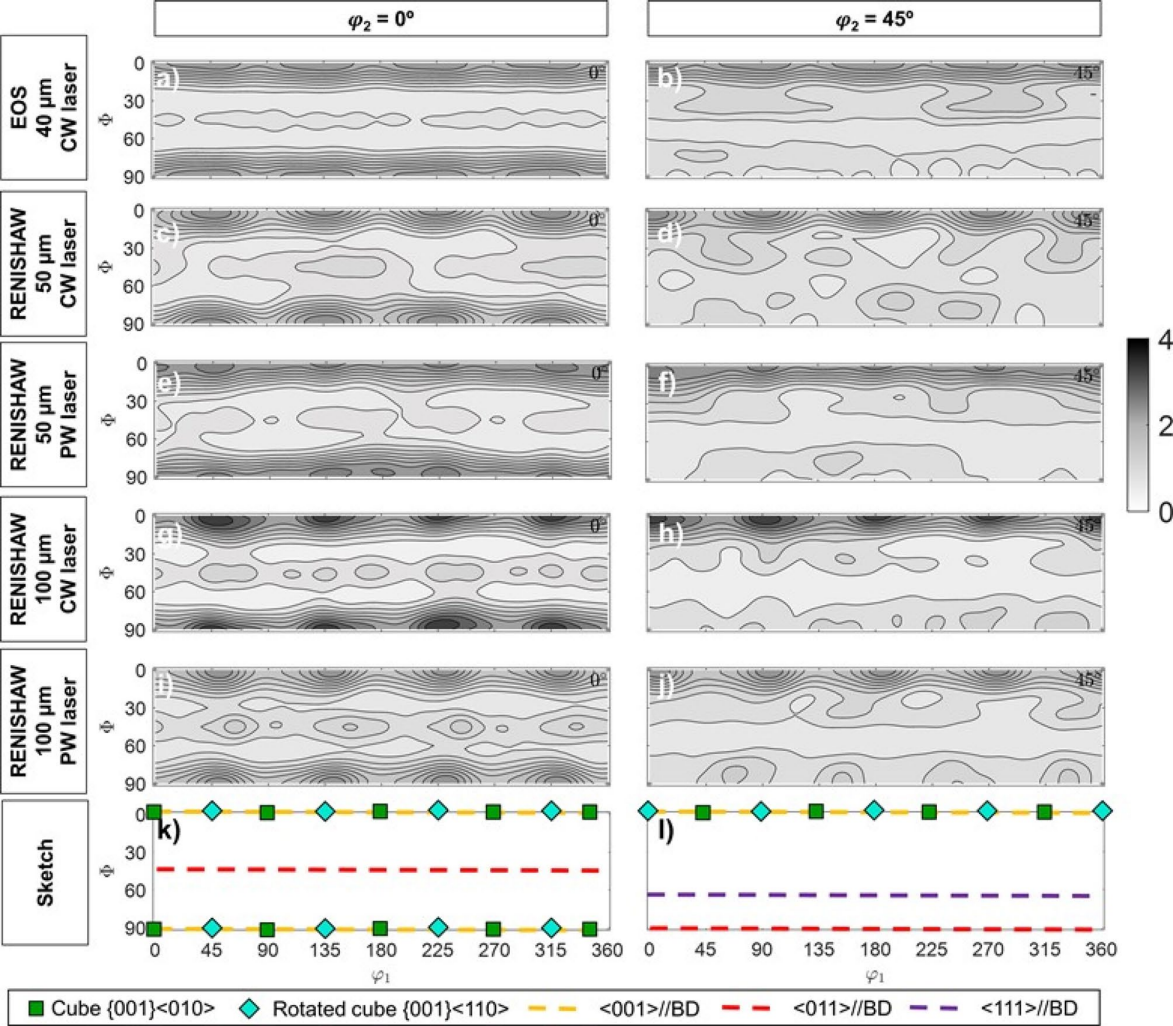
(**a**,**c**,**e**,**g**,**i**,**k**) $\varphi_{2}$ = 0° and (**b**,**d**,**f**,**h**,**j**,**l**) $\varphi_{2}$ = 45° ODF sections corresponding to the martensitic matrix of the top layer of the samples (**a**–**j**) and to a sketch depicting some important texture components or textures that are named in the main text (**k**,**l**). Data corresponds to (**a**,**b**) EOS—40 μm—CW laser; (**c**,**d**) RENISHAW—50 μm—CW laser; (**e**,**f**) RENISHAW—50 μm—PW laser; (**g**,**h**) RENISHAW—100 μm—CW laser and (**i**,**j**) RENISHAW—100 μm—PW laser. Intensities correspond to the color bar on the right-hand side, where the units are multiples of random distribution (MRD).

Subsequently, XRD texture measurements were performed to evaluate the effect of the mentioned processing parameters on the $\alpha ^{\prime}$ texture. Figure 2 shows the $\varphi_{2}$ = 0° and $\varphi_{2}$ = 45° ODF sections corresponding to the martensitic matrix on the top layer of the samples of this study. As can be observed, all of them are characterized by rotated cube {0 0 1} $\langle {1\;1\;0} \rangle$ texture components, where the maximum intensity is never higher than 4 MRD. In some cases, e.g. the EOS condition, the rotated cube texture component seems to have evolved into a $\langle {0\;0\;1} \rangle$//BD fiber, which does not correspond to the initially measured incomplete PF. Similarly, in some cases, e.g. RENISHAW—100 μm, a very weak $\langle {0\;1\;1} \rangle$//BD is present in the ODF sections. No evident effect of the processing parameters, in the ranges studied in this work, is observed.

**Figure 3:**
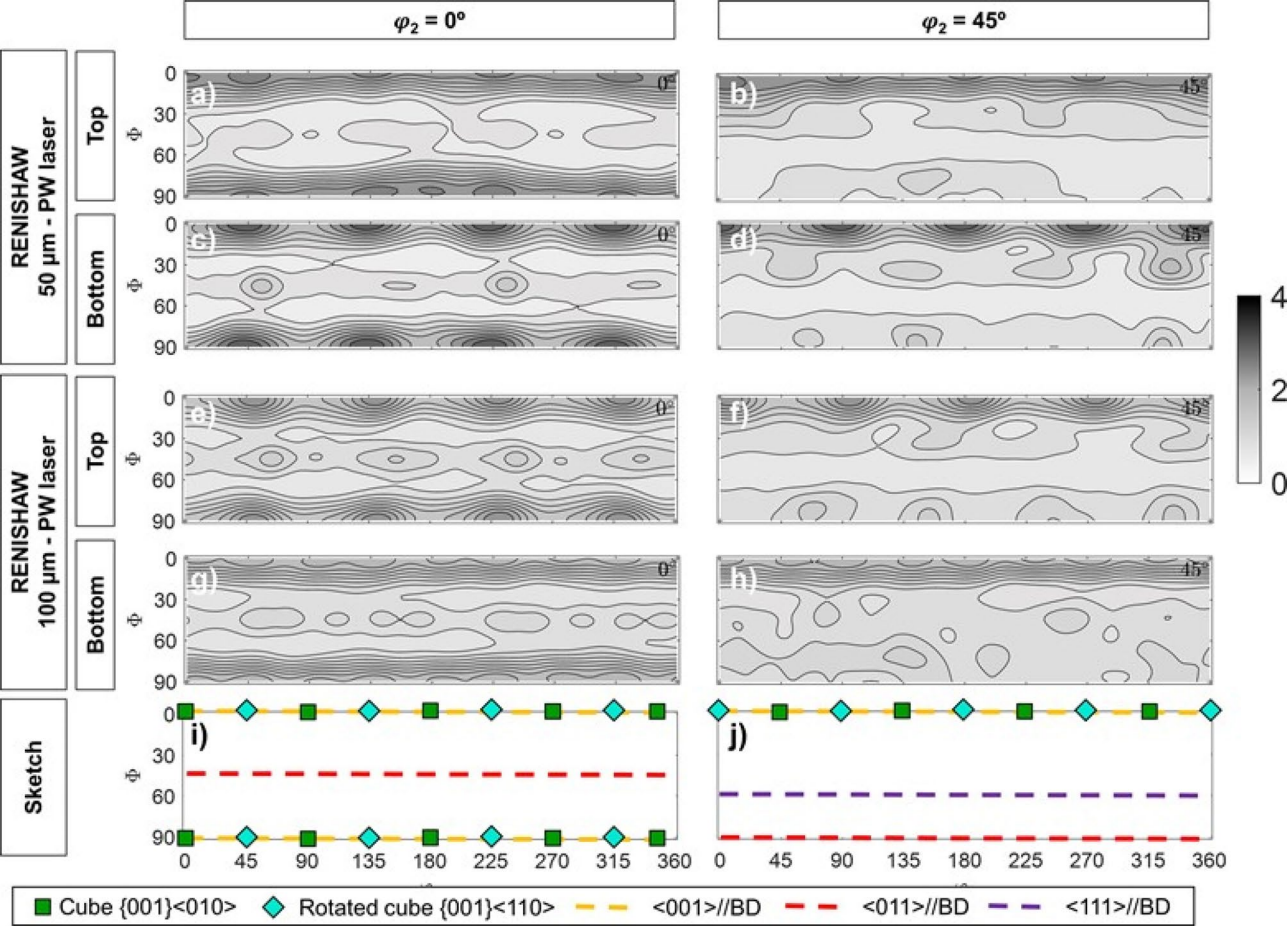
(**a**,**c**,**e**,**g**,**i**) $\varphi_{2}$ = 0° and (**b**,**d**,**f**,**h**,**j**) $\varphi_{2}$ = 45° ODF sections corresponding to the martensitic matrix of the top (**a**,**b**,**e**,**f**) and bottom (**b**,**d**,**f**,**h**) layers of the samples and to a sketch depicting some important texture components or textures that are named in the main text (**i**,**j**). Data corresponds to (**a**–**d**) RENISHAW—50 μm—PW laser and (**e**–**h**) RENISHAW—100 μm—PW laser. Intensities correspond to the color bar on the right-hand side, where the units are multiples of random distribution (MRD).

To evaluate the variation of the texture with reheating cycles, during the printing process, Fig. 3 includes the $\varphi_{2}$ = 0° and $\varphi_{2}$ = 45° ODF sections corresponding to the martensitic matrix of the bottom and top layers of the samples of this study. Note that all conditions presented a similar trend, although only two of them are shown here, for the sake of simplicity. As can be seen, the texture variation is not very pronounced, i.e. the maximum intensity values remain similar regardless of the layer, reason why any variation could be considered in the range of the error bar of the equipment and the ODF calculation.

**Figure 4:**
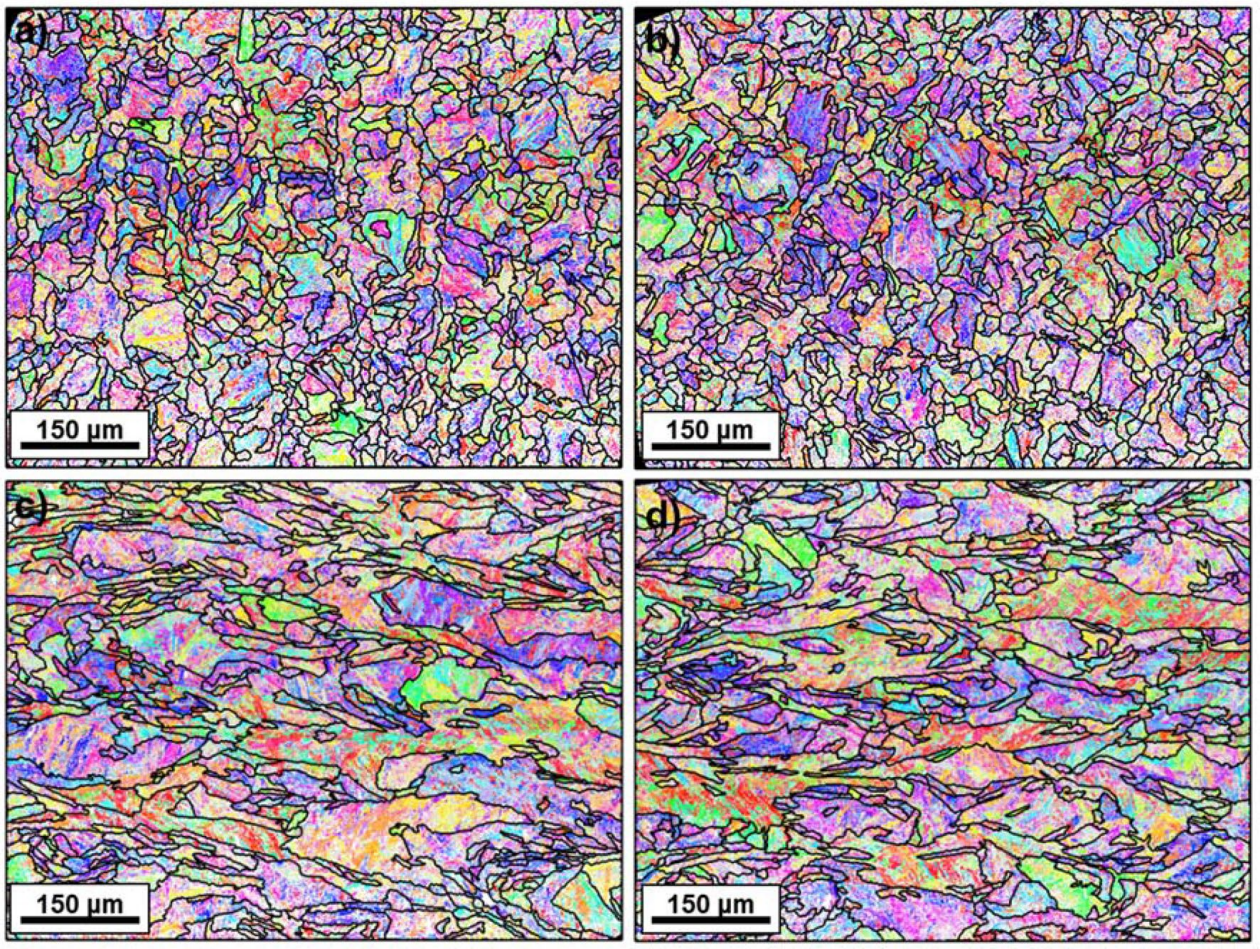
Martensite EBSD maps of the EOS—40 μm—CW laser condition, taken on the (**a**,**b**) transverse section and (**c**,**d**) longitudinal section. Pixels are colored according to their Inverse Pole Figures (IPF) color, corresponding to the building direction—perpendicular to the map for (**a**,**b**) and horizontal for (**c**,**d**). Prior austenite grain boundaries are superimposed on the maps.

**Figure 5:**
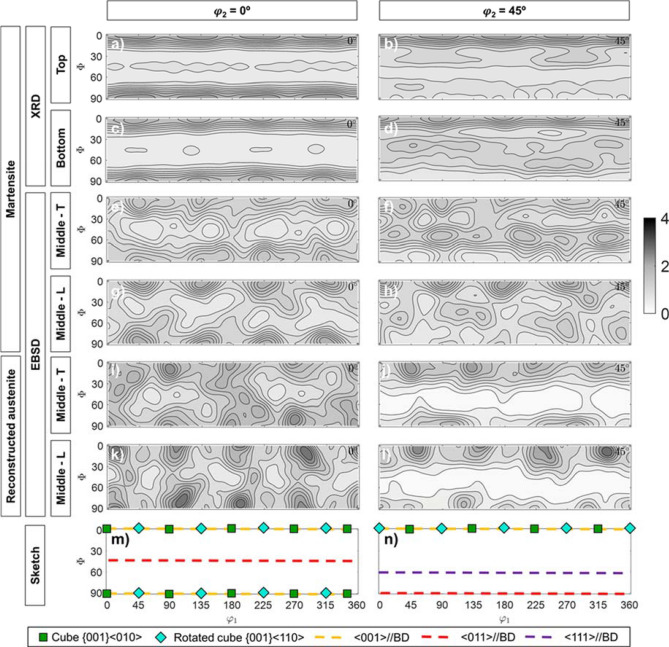
(**a**,**c**,**d**,**g**,**i**,**k**,**m**) $\varphi_{2}$ = 0° and (**b**,**d**,**f**,**h**,**j**,**l**,**n**) $\varphi_{2}$ = 45° ODF sections corresponding to the martensitic matrix of the middle (**e**–**l**), top (**a**,**b**) and bottom (**c**,**d**) layers of the samples and to a sketch depicting some important texture components or textures that are named in the main text (**m**,**n**). Data corresponds to martensite data measured on the EOS—40 μm—CW laser condition by (**a**–**d**) XRD and (**e**–**h**) EBSD (transverse T and longitudinal L sections) and to reconstructed austenite data obtained from the EBSD data (**i**–**l**). Intensities correspond to the color bar on the right-hand side, where the units are multiples of random distribution (MRD).

The XRD texture characterization was followed by a more detailed EBSD characterization of the EOS—40 μm—CW laser condition, which was considered to be representative of all studied conditions. Maps, scanned in the middle transverse and longitudinal sections of the samples, are shown in Fig. 4. Figure 5a–h and m,n compare the $\varphi_{2}$ = 0° and $\varphi_{2}$ = 45° ODF sections corresponding to the EBSD maps to the ODF from measured XRD data for the same condition, where it can be confirmed that textures are similar in terms of maximum intensity components, with the exception that $\langle {0\;0\;1} \rangle$//BD and $\langle {0\;1\;1} \rangle$//BD fibers were only detected by XRD, while a $\langle {1\;1\;1} \rangle$//BD fiber was observed on the transverse section by EBSD. Note that the penetration of X-Rays in Fe structures is of about 30–50 µm, while EBSD only enables to analyze the sample surface, meaning that only one layer is analyzed per condition, except when the longitudinal section was EBSD scanned. In addition, as previously mentioned, initial incomplete PF measured by XRD did not show any fiber. The reason of the presence of these fibers is probably due to the uncertainty in the ODF calculation, given the low intensities of the measured incomplete PF.

The EBSD areas were reconstructed by the algorithm developed by Nyyssönen et al.^21^, which also enabled to determine the experimental orientation relationship (OR) by refining the Kurdjumov–Sachs (K.S) OR, i.e. $\langle { 0.18\; 0.18\; 0.97} \rangle$ 42.85°^22^. The experimental OR was proven to be $\langle {0.{223}\;0.00{2}\;0.{975}} \rangle$ 44.33°, rather far from the most common theoretical OR: the misorientation between the determined experimental OR and the OR defined by Nishiyama–Wassermann (NW)—i.e. $\langle { 0.2 \;0.08 \;0.98} \rangle$ 45.98°^23,24^, KS and Greninger-Troiano (GT) OR, i.e. $\langle { 0.12 \;0.18 \;0.98} \rangle$ 44.26°^25^, were 4.20°, 5.15° and 3.94°, respectively. Figure 4 includes the prior austenite grain boundaries, considering a 10° threshold, where it can be observed how prior austenite grains grew epitaxially along the BD, having lengths of hundreds of micrometers in some cases. This grain morphology is related to epitaxial growth, the growth mode observed in rapid solidification structures, such as the ones of this study^26–32^. Although grain sizes are not homogenously distributed along the area of study, their size is apparently not related to their position with respect to the melt tracks, considering that hatch spacing and layer thickness values lie in the range 50–100 µm. The texture of the reconstructed austenite is shown in Fig. 5i–l and m,n, where it can be seen that, regardless of the section, a cube {0 0 1}$\langle {1\;0\;0} \rangle$ texture component predominates.

**Figure 6:**
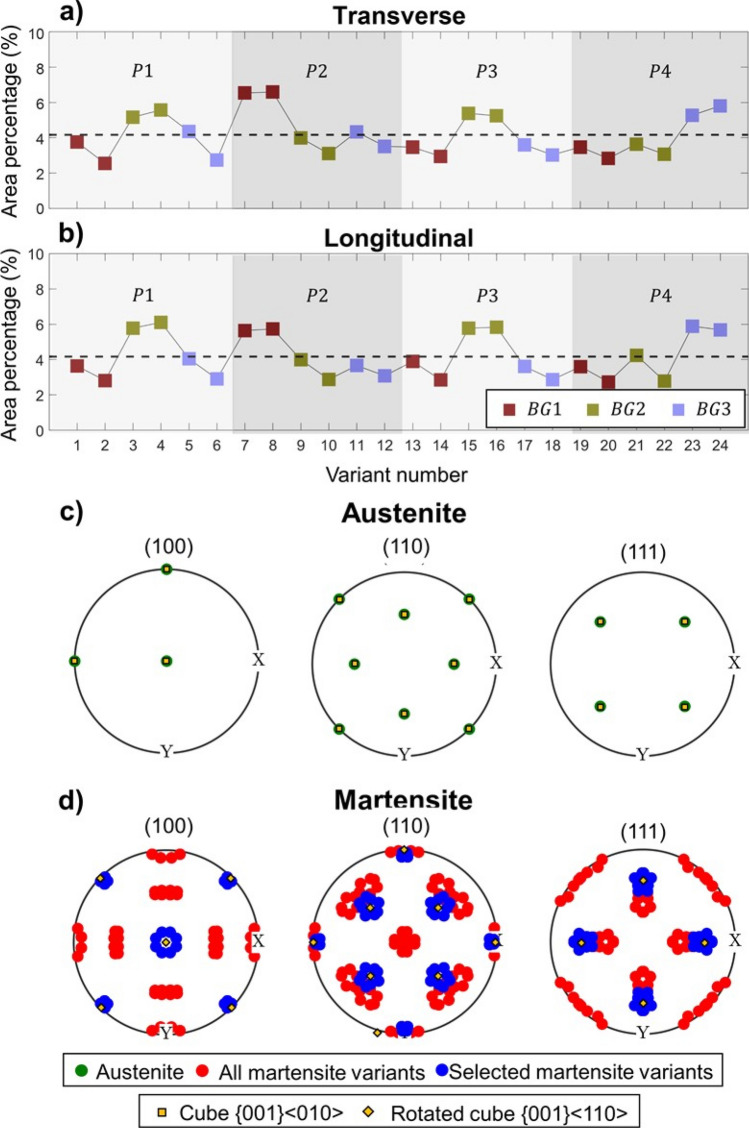
Variant selection study, where (**a**,**b**) represent the area percentages corresponding to each variant number for the EOS—40 μm—CW laser condition. Data corresponds to the EBSD maps taken on the (**a**) transverse section and (**b**) longitudinal sections. Dashed lines represent the area percentage that would be expected without variant selection. The grey areas show which packets the variants belong to, whereas the marker colors change depending on the $BG$ to which the variant belongs. Subfigures (**c**,**d**) show the Theoretical pole figures (PF) corresponding to (**c**) the prior austenite and to (**d**) the resultant martensite, calculated by applying the orientation relationship corresponding to all variants (in red) or only the selected variants (in blue). PFs correspond to the BD.

To study variant selection, the area percentages of each of the variants of the experimental OR were estimated. In order to make variant indexation consistent with the global reference system, the austenite orientations were redefined so that the BD axis was contained in the triangle delimited by the directions $[ {0 \;0\; 1} ]$ − $[ {\overline{1}\; 1\; 1} ]$ − $[ {0 \;1 \;1} ]$. Even though this methodology has been previously used in the past when trying to correlate variants to external prior austenite deformation^33,34^, it can be a systematic way of indexing variants from different prior austenite grains. Figure 6a,b includes the quantified area percentages for each variant. Variants are divided into different packages and Bain groups, while consecutive pairs of variants belong to the same block. This division was performed to assess whether there is any relationship between their selection and their belonging to a given package, block or Bain group. As can be observed, even though variant selection is not very strong in any case, regardless of the section of study, variants 3, 4, 7, 8, 15, 16, 23 and 24 show a slightly higher area percentage. These variants belong to different packages and Bain groups, although they are always pairing variants belonging to the same crystallographic block.

To assess the contribution of these selected variants to the macro-texture, the theoretical textures that would be expected if all variants vs. only the selected variants formed, given the measured prior austenite texture, were calculated and are included in Fig. 6c,d. To simplify the calculation, the austenite texture was assumed to only consist of a cube component, {0 0 1}$\langle {1\;0\;0} \rangle$. As can be observed, selected variants are those that lie as close as possible to a martensitic rotated cube texture, i.e. the misorientation angle between the selected variants and a cubic rotated orientation is 9.8°.

**Figure 7:**
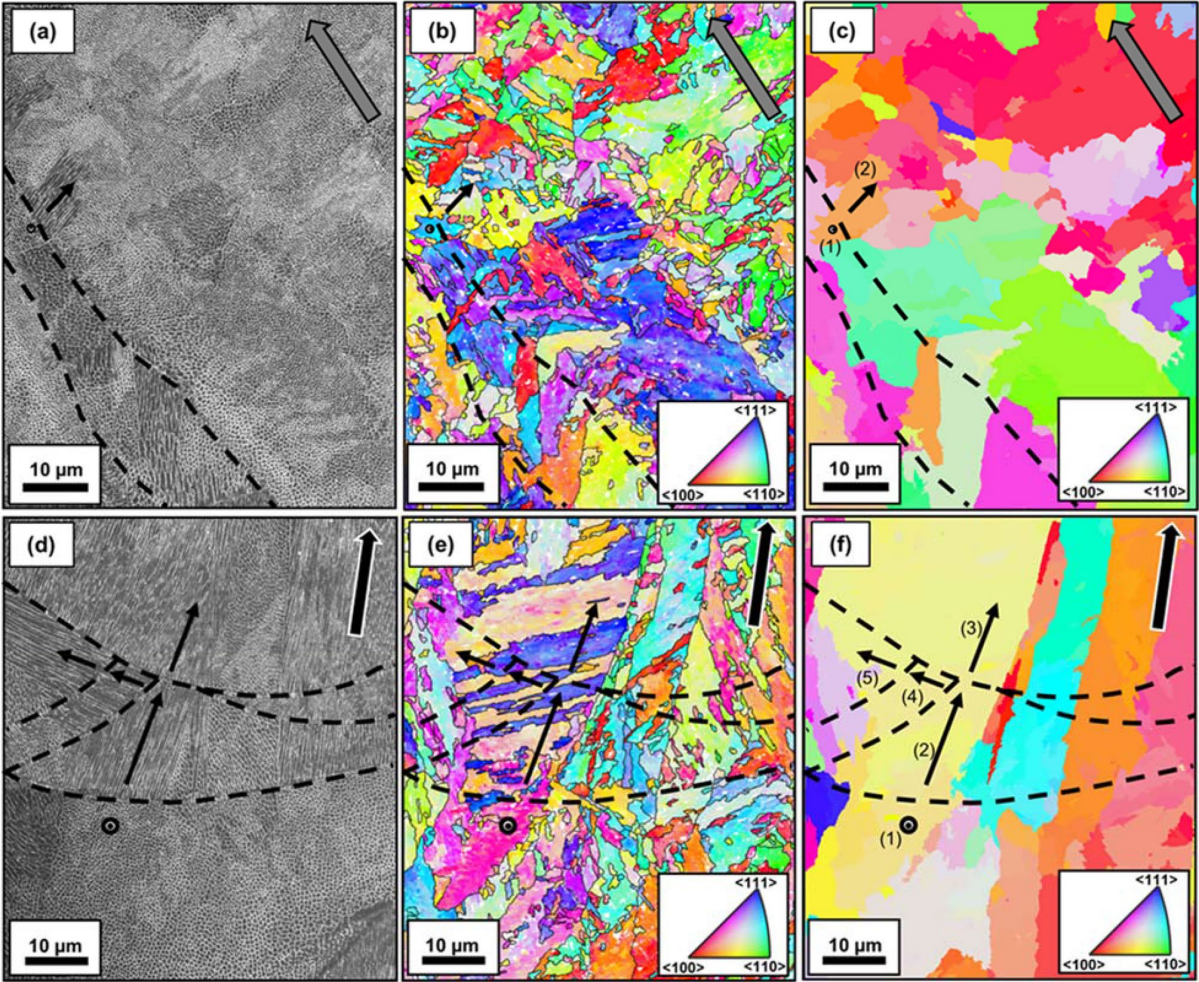
Correlative (**a**,**d**) SEM (**b**,**c**,**e**,**f**) EBSD results corresponding to the transverse (**a**–**c**) and longitudinal (**d**–**f**) sections of the EOS condition—40 μm—CW laser, where the SD and the BD are indicated by a grey and a black arrow, respectively, and where the EBSD orientations are colored according to their IPF—BD color. The EBSD data correspond to (**b**,**e**) the bcc phase and (**c**,**f**) the corresponding reconstructed prior fcc phase. The dashed black lines represent the MP boundary and the arrows represent the different colony growth directions in given prior fcc grain. Each of the arrows is identified by an ID.

Finally, the microstructure was studied in detail at higher magnification. Figure 7 includes the correlative SEM-EBSD study for the reference EOS condition, corresponding to the transverse and longitudinal sections, respectively. Both images include an SEM micrograph (Fig. 7a,d), its corresponding bcc and reconstructed prior fcc (austenite) Inverse Pole Figure (IPF) maps (Fig. 7b,c and e,f), where the IPF coloring corresponds to the BD. No retained fcc map is shown as the fcc phase was barely indexed, i.e. only 0.01 and 0.08% were indexed on the transverse and longitudinal sections, respectively. The low indexation is due to the size of the fcc features, which makes Kikuchi pattern indexation more complex, given the beam spot size. With regards to the figures, while the BD is perpendicular to the transverse section in Fig. 7a–c, it is indicated by an arrow on the top right side of the subfigures in Fig. 7d–f. Additionally, the MP boundaries are highlighted by thick black dashed lines and the bcc sub-block boundaries (defined as areas where the misorientation angle values were lower than 6°) are represented by thinner black solid lines.

Both correlative SEM-EBSD analyses are useful to understand the printing process. With respect to the transverse section, Fig. 7a shows two different MP boundaries, which implies that the cutting section was below the MP overlap region. Also, it is shown how a cellular colony grows perpendicular to the right MP boundary towards the MP on the right-hand side, suggesting that this MP was formed after the one on the left-hand side. The fact that cellular colonies tend to grow as perpendicular as possible to the MP boundary (along the heat flux)^35^, suggests that the left MP was re-melted during the printing process. With respect to the prior fcc grains, Fig. 7c evidences, once again, the epitaxial growth of the prior austenite grains across the MP boundary, where most of the prior fcc grains have irregular shapes and their size does not vary when approaching the MP boundary. In the highlighted case, the cellular colony growth direction does not keep its growth direction when crossing the MP boundary, but it apparently rotates by 90°.

Regarding the longitudinal section, prior fcc grains are elongated due to their epitaxial growth along the direction of maximum heat extraction, approximately parallel to the BD, as can be observed in Fig. 7f, where some grains stopped growing when encountering another grain, i.e. competitive growth. Moreover, in some cases, the cellular colony growth direction does not keep its growth direction when crossing the MP boundary, but it apparently rotates by 90° on the plane of observation.

To further study this phenomenon, several colonies were selected from Fig. 7a,d, where all the colonies in a given map belong to the same prior fcc grain, i.e. the orange prior fcc grain in Fig. 7c and the yellow prior fcc grain in Fig. 7f. The growth directions of these colonies were identified on the micrographs in Fig. 7a,d and depicted by black arrows. Based on the morphology of the cell boundaries and according to previous works, it was assumed that the colony growth directions were either perpendicular or parallel to the studied section^36^. However, it is noteworthy to mention that there could be small misalignments that could affect the subsequent calculation.

The prior fcc crystallographic directions that correspond to the drawn arrows were calculated from the EBSD data and included in Table 1. Moreover, the minimum angles in between the calculated crystallographic directions and the directions belonging to the $\langle {1\; 0 \;0} \rangle$ family were calculated. As can be seen, all the minimum angles with respect to the $\langle {1 \;0 \;0} \rangle$ family are lower than 16.5°. With respect to the remaining conditions, they also presented epitaxial growth. Moreover, this phenomenon, were colonies rotate by 90°, has been observed on all the studied conditions along the whole microstructure, find SEM micrographs in Supplementary Material C.

**Table 1:** For the regions identified in Fig. 7 by an ID, colony growth direction and minimum angle between the corresponding colony growth direction and the prior fcc directions belonging to the family $\langle {1 \;0 \;0} \rangle$.

Section	ID	Colony growth direction	Minimum angle between colony growth direction and the directions $\langle {1{ }\;0\;{ }0} \rangle$ (°)
T	1	$[ {0.06\;{ }\overline{0.27} \;{ }0.96} ]$	16.4
	2	$[ {\overline{0.01} { }\;0.96{ }\;0.28} ]$	16.1
L	1	$[ {\overline{0.10} \;{ }0.01{ }\;0.99} ]$	5.9
	2	$[ {\overline{0.96} \;{ }0.26\;{ }\overline{0.01} } ]$	16.4
	3	$[ {\overline{0.96} { }\;0.26\;{ }\overline{0.01} } ]$	16.4
	4	$[ {\overline{0.28\;} \overline{0.96\;} { }\overline{0.02} } ]$	16.1
	5	$[ {\overline{0.25} \; \overline{0.97} \;{ }0.01} ]$	14.4

Finally, regardless of the section, it can be seen that the bcc phase transformed from the prior fcc phase, forming sub-blocks that sometimes crossed the MP boundaries, as can be seen in Fig. 7b,e.

## Discussion

### Effect of the processing parameters on the resultant texture

The first of the questions to be answered in this work is what the effect of the thermal gradient and solidification velocity, i.e. the processing parameters, on the prior austenite and the martensite textures is. This work suggests that the effect of the printer, the layer thickness and the laser emission mode on the texture of the martensitic matrix is not significant for the studied conditions. Martensite texture remained unaltered, i.e. showing rotated cube {0 0 1}$\langle {1\;1\;0} \rangle$ texture components, regardless of the condition. Because the martensite texture did not significantly vary, it is fair to assume that neither did the prior austenite texture, which has a cube {0 0 1}$\langle {1\;0\;0} \rangle$ texture. Note that larger variations of layer thicknesses or a different laser emission strategy or printer could still lead to texture variations, for which a systematic study, including different parameters in larger ranges, is needed. However, it is expected that the effect of these parameters is not as significant as the effect of power or laser speed. Given that cellular colonies grow as perpendicular as possible to the MP boundary (along the heat flux)^35^, it is fair to assume that melt pool shape directly affects texture. The literature concerning the effect of the layer thickness on the MP dimensions is still scarce. Experimental results show some discrepancies, as some authors have concluded that thicker powder layers lead to slightly smaller MPs^37^, whereas some other authors have observed the opposite behavior^38^. Simulations agree with the latter results, as they point out that thicker layer thicknesses lead to higher peak temperatures^39^ because the powder has a lower thermal conductivity than the bulk material solidified in bottom layers^40,41^. The effect of layer thicknesses on MP shape is even less clear. Also, the laser emission mode can affect the temperature evolution in a given MP and, thus, its shape and dimensions. For a fixed laser power, the MP peak temperature obtained for a CW laser is similar to the one obtained for a PW laser during the pulse^42^. However, the temperature has been shown to drastically drop during the jump delay in the case of the PW laser^43^. In addition, changing the laser emission mode has also been associated to modifying the MP shape^44^. Therefore, theoretically, MPs obtained by the CW laser mode should be more elongated along the SD than the MPs obtained by the PW laser.

As previously mentioned, Kannan and Nandwana^16^ reported that the martensitic microstructure formed by subjecting a 300 maraging steel to LPBF with an unknown scan rotation strategy, a power laser of ~ 110 W, a scan speed of ~ 1500 mm/s, a hatch spacing of ~ 50 µm and a layer thickness of ~ 45 µm did not present any predominant texture component or fiber in the as-built condition. They correlated these results with previous studies^13,14^ with different laser power, speed, hatch spacing and layer thickness and a rotation strategy of 90°, although both studies based their conclusions on small area EBSD scans. Kannan and Nandwana^16^ also reported negligible prior austenite texture, in disagreement with the results obtained in this work. It is possible that their low texture indexes in the prior austenite (maximum ODF intensities < 2 MRD) were related to their scan strategy, laser power or laser speed. Note that stronger textures can be typically found at higher energies^45,46^. The low texture in the prior austenite could then be inherited by the martensite after the phase transformation. Although we cannot conclude what the effect of each of these processing parameters is, the comparison of Kannan and Nandwana’s results^16^ to our results evidences that prior austenite texture can be changed by modifying laser power, laser speed or scan strategy.

Finally, the study of variant selection has proven that there are some predominant variants that always belong to the same blocks, although their area percentage is not very high as compared to the rest of them, in good agreement with Kannan and Nandwana^16^. It has been found that the selected variants are those that contribute to a martensite cubic rotated texture the most.

### Effect of the thermal flux and the heat treatment intrinsic to the LPBF process on the prior austenite texture

Once the effect of the processing parameters on the as-solidified texture has been evaluated, one can proceed to study what the relationship of this observed texture and the heat flux is. It is important to acknowledge that colonies have shown to lie nearly parallel to the $\langle {1 0 0} \rangle$ family of directions, in good agreement with previous results on austenitic steels^47,48^. This phenomenon is characteristic of dendrites^49,50^, therefore, it is possible that the observed cells were dendrites instead, where their secondary arm spacing was so small that it was not observable. Moreover, colonies underwent a change in the colony growth direction, i.e. side-branching, where the new branch is rotated by 90° with respect to the oldest one^51^. For instance, it can be observed that the direction $[ {\overline{0.96} { }\;0.26\;{ }\overline{0.01} } ]$ found in colony (3) in Fig. 7f changed to $[ {\overline{0.28} \;{ }\overline{0.96} \;{ }\overline{0.02} } ]$ once crossing the MP boundary towards colony (4). Side-branching happens when the local thermal gradient in a new MP is not parallel to the colony growth direction in the MP below. In LPBF microstructures, side branching has been reported to lead to grain coarsening and to promote helical epitaxial growth, especially when layers are rotated by an angle of 67° with respect to each other, where texture fibers could appear^51^. Therefore, the observed side-branching is expected to have affected the texture of the as-solidified microstructure, before any thermal cycle happened.

With respect to the effect of thermal cycling, results showed that the martensite texture in the bottom layer (rotated cube {0 0 1}$\langle {1\;1\;0} \rangle$ texture)—layer subjected to a cyclic heat treatment as subsequent layers were deposited on top of it—did not significantly change with respect to the top layer, layer that was not so affected by the heat. It can be thus assumed that the prior austenite texture did not change during the process either. Note that the microstructure in the top layer was still affected by the heat associated to the deposition of subsequent tracks on the same deposition layer. However, the fact that texture did not significantly change as a function of the layer height suggests that the heat dissipated by subsequent laser tracks would not affect texture either. A more thorough study to evaluate the effect of the deposition of subsequent track melts on the microstructure would imply melting two isolated subsequent tracks. Hence, these results suggest that the prior austenite was not recrystallized during the process, as Kannan and Nandwana^16^ proposed.

The high magnification characterization can help to further confirm this finding. Figure 4a,b shows how most of the prior fcc grains were irregularly shaped and no refinement near the MP boundary was detected, oppositely to what has been reported in the literature for other alloys^30^. Prior fcc grains grew elongated, approximately parallel to the BD, as can be observed in the longitudinal section (Fig. 4c,d). Moreover, it is interesting to see how, in this work, the parallelism between the colony growth direction and the $\langle {1 \;0 \;0} \rangle$ family of directions was kept, despite the fact that the microstructure had been re-austenitized when subsequent layers were deposited and melted on top of it. It is important to mention that the re-austenitized area is not expected to have been large, in good agreement with previous studies in which similar laser power and speed were used^52^, and only a small region should have been subjected to temperatures above the steel ferrite-to-austenite critical temperature, Ac1. Thus, considering the pronounced elongation of most of the prior fcc grains, the possibility of every prior fcc grain forming at once through recrystallization during the process is ruled out. The retention of austenite and its subsequent growth during the cyclic treatment to which the structure is subjected during the LPBF process could explain the fact that the parallelism between the prior fcc $\langle {1 \;0 \;0} \rangle$ directions and the cellular growth directions is kept. Austenite reversion happening during the LPBF process has already been reported in the past^53^, although not in the same terms of this work. The obtained results suggest that, when an already solidified layer (with a low fraction of retained austenite) is re-austenitized by the redeposition of material, the fcc structure keeps the same initial crystallographic orientation. However, further investigations are needed to clarify the transformation mechanism by which re-austenitized austenite inherits the same crystallographic orientation of the original prior austenite.

## Conclusions

1. Laser power, laser speed and scan strategy can affect the resultant texture in 300 maraging steels, although the separate effect of each of them still has to be discovered. For the studied ranges, the effect of the printer, the layer thickness or the laser emission mode is negligible. Whether larger variations of layer thicknesses or a different laser emission strategy or printer could still lead to texture variations remains as an open question that must be further studied.
2. Weak variant selection phenomena have been identified, where selected variants always belong to the same crystallographic blocks. No relationship between the selected variants, their pertinence to crystallographic packets or Bain groups has been observed, although it has been found out that selected variants are those that contribute to a martensite cubic rotated texture the most.
3. The observed prior austenite texture is due to the heat flux, which promotes side-branching phenomena for the 67° scan rotation strategy. The prior austenite grows through a competitive and epitaxial mechanism, where no recrystallization happens during subsequent thermal cycling. During this process, austenite transforms to martensite, although a small fraction of austenite is retained. When the structure is reheated because of a subsequent deposition, the re-austenitized grains keep the same crystallographic orientation as the surrounding retained austenite. Further investigations are needed to understand this phenomenon.

## Supplementary Information

Supplementary Information.

## Data Availability

The datasets generated during and/or analyzed during the current study are available from the corresponding author on reasonable request.
