# Supplementary material for: Effect of processing parameters on texture and variant selection of as-built 300 maraging steel processed by laser powder bed fusion
Source: Sci Rep. 2022 Sep 28;12:16168. doi: 10.1038/s41598-022-19835-9 (PMC9519872; doi:10.1038/s41598-022-19835-9)
Supplement: Supplementary file 1 — Supplementary Information. [file 41598_2022_19835_MOESM1_ESM.docx]

Effect of processing parameters on texture and variant selection of as-built 300 maraging steel processed by laser powder bed fusion

Adriana Eres-Castellanos, Ana Santana, David De-Castro, Jose Antonio Jimenez, Rosalia Rementeria, C. Capdevila and Francisca G. Caballero

**Supplementary material**

1. **Steel chemical composition**

In this work, commercial Maraging 300 powder was used to build parts by LPBF. Parts built in Maraging 300 have a chemical composition corresponding to the United States classification 18% Ni Maraging 300. The steel chemical composition, relative density and density are included in Table 1. While the weight percentages of C, S, N and O were determined by LECO, the percentages of the remaining elements were estimated by Inductively coupled plasma-optical emission spectroscopy (ICP-OES).

Table 1. Steel compositional, relative density and density of the commercial Maraging 300 powder used to build parts in this work

| Chemical composition (wt. %) | | | | | | | | | | | | | | Relative Density (%) | Density (g/cm^3^) | |
| --- | --- | --- | --- | --- | --- | --- | --- | --- | --- | --- | --- | --- | --- | --- | --- | --- |
| Ni | Co | Mo | Ti | Cr | Si | Al | O | Mn | P | C | S | N | Fe |  |  |  |
| 18 | 9.3 | 4.85 | 1.08 | 0.15 | 0.13 | 0.12 | 0.11 | 0.03 | 0.007 | 0.007 | 0.004 | <0.001 | bal. | ≈ 100 | 8.0-8.1 |  |

1. **Printing specifications**

A simplified representation of the evolution of the power and distance increase along a track (ΔDistance) as a function of time for the conditions of study can be seen in Figure 1(a-c). The hatch strategy of an individual layer consisted of a meander pattern, where successive layers were rotated by a 67º angle, as the sketch in Figure 1(d) shows. Figure 1(e) shows the dimensions of the parts built with the RENISHAW printer, where the sections of study are highlighted.


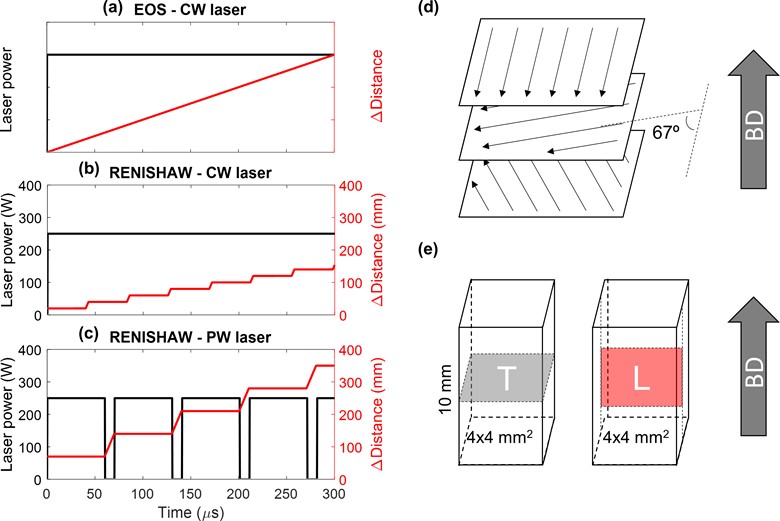


Figure 1. Simplified representation of (a-c) laser power and distance increase along the track (ΔDistance) as a function of time for the conditions (a) EOS – CW laser; (b) RENISHAW – CW laser and (c) RENISHAW – PW laser; (d) sketch showing the printing strategy used in this work; (e) sketch showing the sections to characterize in this work: transverse (T) and longitudinal (L). BD stands for building direction.

1. **Side-branching**

Because a detailed correlative SEM-EBSD study, such as the one included in the main draft (in which the crystallographic directions corresponding to certain cellular colonies at both sides of the melt pool boundary are calculated) can be very time consuming, we have only performed it for one printing condition in both, transverse and longitudinal sections. However, SEM micrographs from all the printing conditions used for study confirm that colonies rotate by 90º at the melt pool boundary in multiple cases, for all the conditions of study. In Figure 2, we include some micrographs, where the direction of the colonies is highlighted by red arrows (or a dot, in case the direction is out of the plane). As can be observed, this phenomenon repeats all over the microstructure, regardless of the condition under study.


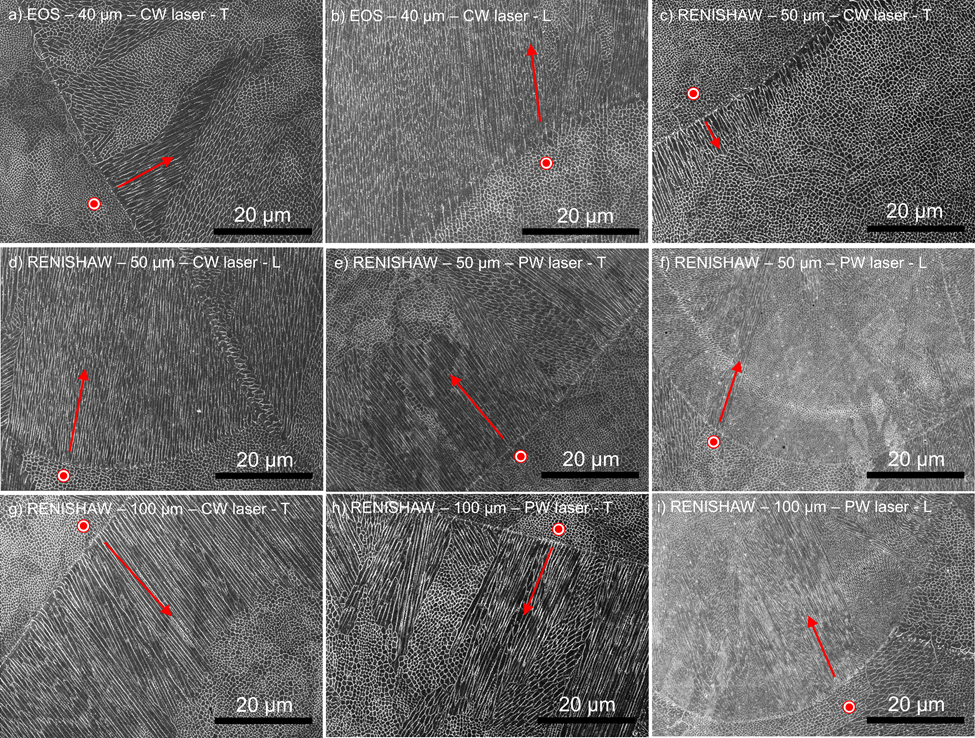


Figure 2. Micrographs exemplifying side branching for all the conditions of study in this work: (a) EOS – 40 μm – CW laser – T; (b) EOS – 40 μm – CW laser – L; (c) RENISHAW – 50 μm – CW laser – T; (d) RENISHAW – 50 μm – CW laser – L; (e) RENISHAW – 50 μm – PW laser – T; (f) RENISHAW – 50 μm – PW laser – L; (g) RENISHAW – 100 μm – CW laser – T; (h) RENISHAW – 100 μm – PW laser – T; (i) RENISHAW – 100 μm – PW laser – L. T and L stand for transverse and longitudinal sections, respectively. Colony directions of growth are represented by red arrows.
